# Clear cell renal cancer metastasis in the contralateral ureter: a case report

**DOI:** 10.1186/s13256-021-02839-w

**Published:** 2021-05-30

**Authors:** Julien Blanc, Beat Roth

**Affiliations:** 1 Department of Urology, Anna-Seiler Haus, Inselspital, University of Berne, CH-3010 Berne, Switzerland; 2 Departement of Urology, Lausanne University hospital and University of Lausanne, Rue du Bugnon 46, 1011 Lausanne, Switzerland

**Keywords:** Urology, Case report, Clear renal cell carcinoma, Ureteral metastasis

## Abstract

**Background:**

Clear cell renal carcinoma is known for its propensity for metastatic spread. Common sites of metastasis are the lungs, bones, lymph nodes, liver, adrenals and brain, but all organs can be affected. Contralateral ureteral metastasis is a rare phenomenon, and only a few cases have been reported in the literature.

**Case presentation:**

We present the case of a 58-year-old Caucasian patient with a single contralateral ureteral metastasis of a clear cell renal carcinoma.

**Conclusion:**

Ureteral metastasis of clear cell renal carcinoma is very rare, and there is no well-established treatment. For patients with low metastatic spread/volume, the aim should be to preserve kidney function, and thus metastasectomy should be considered.

## Background

Renal cell carcinoma (RCC) accounts for 2–3% of all cancers worldwide, and 80% of these are clear cell RCC [1]. In a large population-based analysis from 2012, the most common sites for metastasis were the lungs (45.2%), bones (29.5%), lymph nodes (21.8%), liver (20.3%), adrenals (8.9%) and brain (8.1%) [2], but metastases can occur in virtually all organs [1].

For localized tumors, radical or partial nephrectomy is the standard of treatment, while for advanced or metastatic RCC, a cytoreductive nephrectomy followed by systemic therapy has been the standard of care. A prospective, randomized trial demonstrated the non-inferiority of sunitinib to nephrectomy followed by sunitinib therapy, introducing the possibility for the use of systemic therapy alone without surgery in metastatic disease [3].

Ureteral metastasis of kidney cancer is very rare; only a few case reports have been described in the literature [4–10]. Moreover, ureteral metastasis of RCC is both a diagnostic and therapeutic challenge.

We present a rare case of contralateral ureteral metastasis that was successfully treated with open segmental ureterectomy and end-to-end anastomosis, with good functional and oncological outcomes.

## Case presentation

**Fig. 1:**
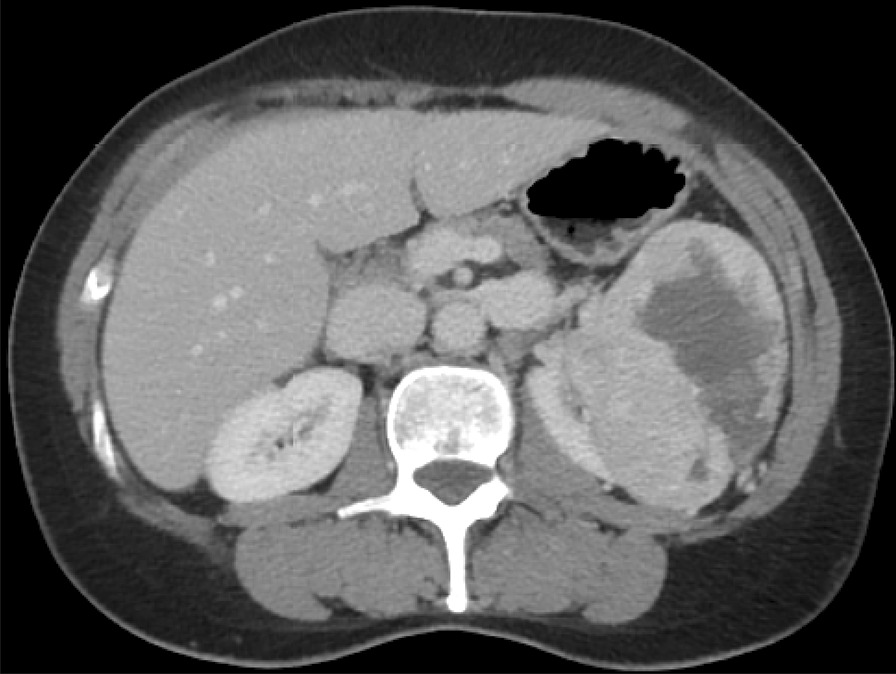
Computed tomography image of the left renal mass at diagnosis.

A 58-year-old Caucasian female patient experienced painless recurring macrohematuria and weight loss (6 kg over a year). Anamnesis revealed an otherwise healthy patient, without previous operations or health problems and currently taking no medications. The patient consumed alcohol only occasionally and had never smoked. Family history was nonsignificant, especially for urological tumor. The patient worked as a commercial employee. On physical examination, no specific findings were noted; in particular, no mass was palpable at the costovertebral angle that was otherwise painless at percussion. Neurological examination revealed no focal deficit. The patient presented no fever (36.5 °C), and blood pressure was in the normal range (128 mmHg), as well as heart rate (78 beats per minute) and oxygen saturation (SpO_2_ 97%). Laboratory results were as follows: creatinine 82 µmol/L, white blood cells 11.1 × 10^9^/L, hemoglobin 122 g/L and platelets 231 × 10^9^/L. At the time of examination, urinalysis showed no pathological findings despite previous macrohematuria. Further investigations with contrast-enhanced computed tomography (CT) showed a renal mass (92 × 68 × 97 mm) in the upper pole and pars intermedia of the left kidney (Fig. 1). In addition, in the excretory phases, a 10 mm wall thickening in the mid-right ureter was discovered. Staging CT of the chest/abdomen/pelvis and bone scintigraphy showed no other suspicious tumor lesions. Because of suspicion of urothelial carcinoma in the right ureter, a diagnostic ureteroscopy including endoscopic biopsies of the region of interest was performed. At the ureteroscopy, the lesion had the appearance of a polypoid, solid, spherical tumor with a vascular pedicle of the ureteral lateral wall. Microscopically, the biopsies revealed light chronic inflammation and granulation tissue with areas of calcification. The final diagnosis, based on the macroscopic and microscopic analysis, was of a polyp with granulation tissue. All biopsies and cytologies from both ureters showed absence of malignancy.

Considering the size of the kidney tumor and after ruling out a urothelial carcinoma, an open surgical approach was chosen with open (11th intercostal space lumbotomy) left radical nephrectomy with adrenalectomy and locoregional lymphadenectomy. Intraoperatively, the tumor had a rich collateral circulation, and at the upper pole a peritoneal window was excised because of suspicion of tumor infiltration. However, with the exception of the adrenal gland, all neighboring organs were spared.

The postoperative period was uncomplicated. In the early postoperative period, the patient had epidural anesthesia with bupivacaine 0.5% that was stopped on day 5 after a relay with acetaminophen 1 g and metamizole orally four times a day, with oxycodone (5 mg) taken only occasionally. The patient was discharged on day 9.

The histopathological examination revealed a clear cell renal carcinoma, maximum diameter of 10.2 cm, with foci of sarcomatoid differentiation, infiltration of the perirenal fat and vascular invasion, and negative margin. The final diagnosis according to the TNM classification was a clear cell renal carcinoma pT3a, pN0 (0/9), V1, L0, Pn0, G3-4, R0.

Given the high-risk profile of the tumor but with complete resection, a close follow-up with CT without adjuvant therapy was chosen.

**Fig. 2:**
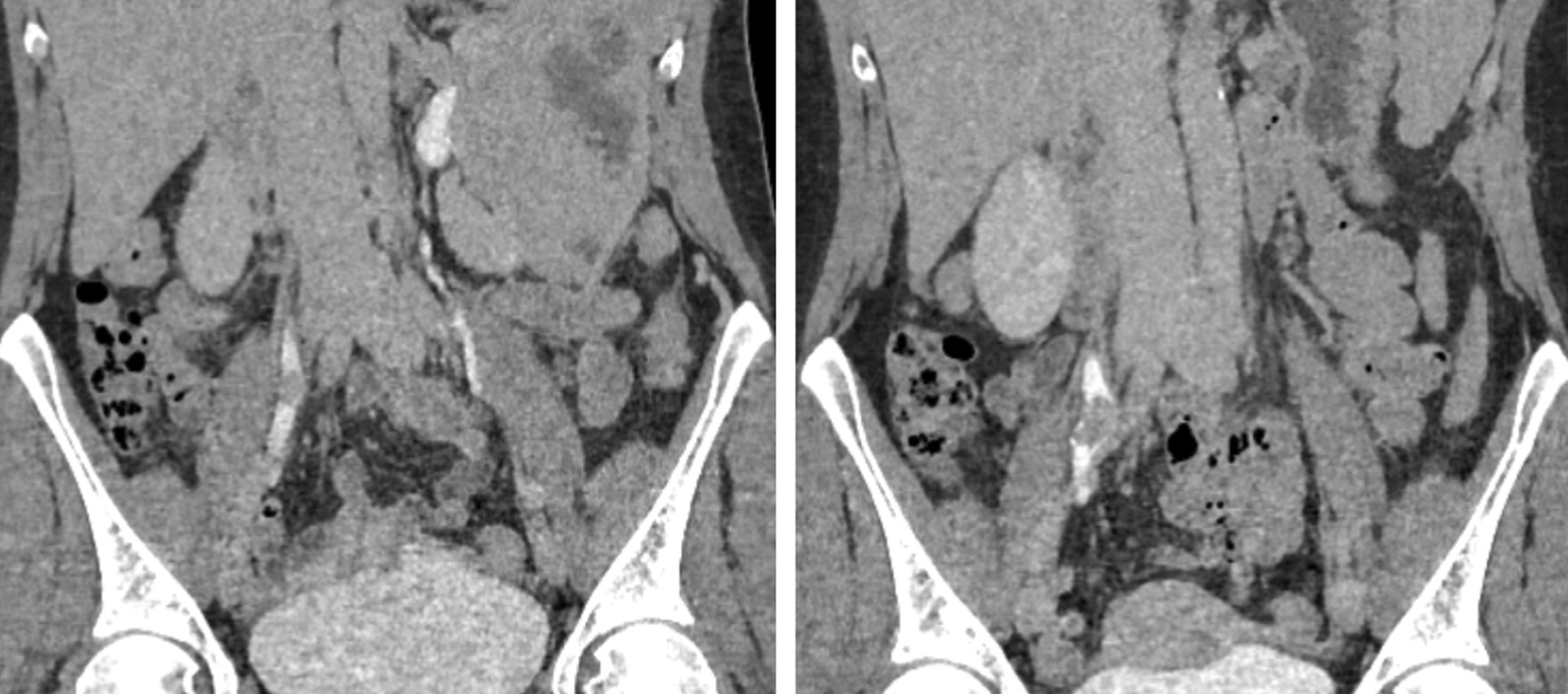
Left: initial appearance of the lesion in the excretory phases. Right: 4-month follow-up.

**Fig. 3:**
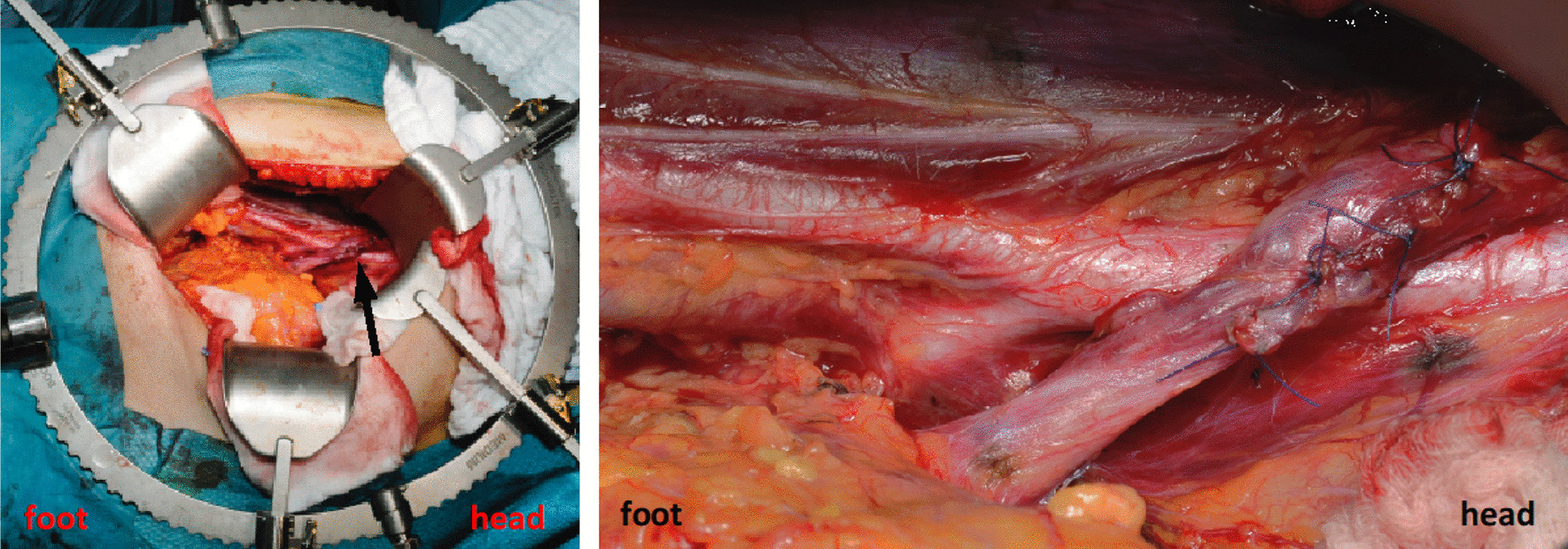
Intraoperative photographs. The black arrowhead shows the end-to-end anastomosis. Right: details of the anastomosis.

At the 4-month follow-up, the patient presented with persisting painless macrohematuria, and follow-up CT of the chest/abdomen/pelvis showed a solid, contrast-enhanced lesion of the right mid-ureter with significant growth (18 mm) in the excretory phases (Fig. 2). Imaging showed no other lesions or lymphadenopathies which could suggest metastases. With regard to an unknown lesion with a significant size progression, with risk of obstruction of the ureter in a single-kidney patient, an open resection of the lesion was chosen. Intraoperative frozen section analysis revealed an invasive urothelial carcinoma with free section margins. Finally, in the absence of other urothelial lesions, a partial ureterectomy with end-to-end anastomosis and locoregional (paracaval, inter-aortocaval, and internal, external and common iliac) lymphadenectomy was performed (Fig. 3).

**Fig. 4:**
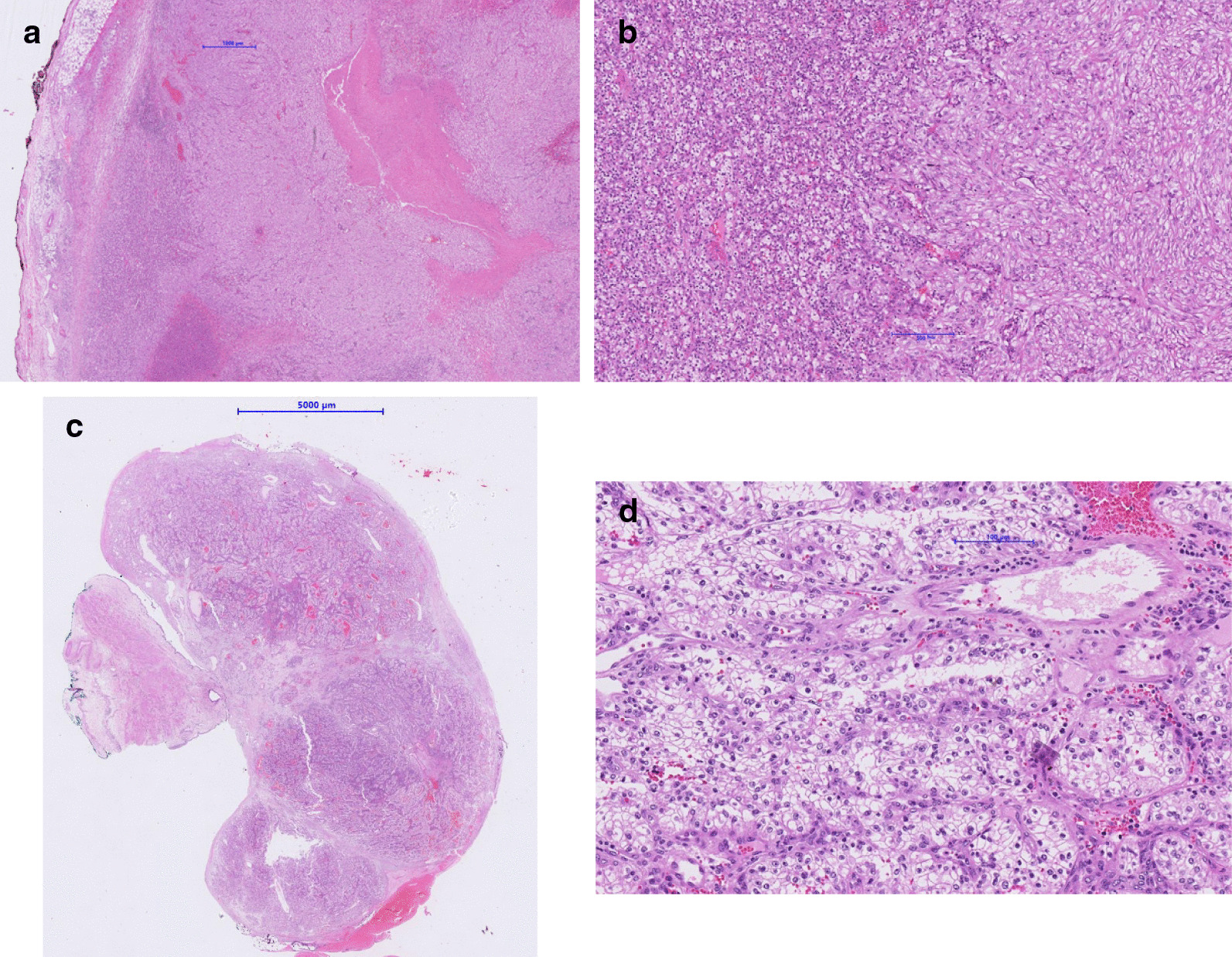
Histological sections. **a** Cross section of tumor renal resection specimen. **b** Renal clear cell carcinoma at higher magnification with clear cell morphology (left) and abrupt transition to sarcomatoid morphology (right). **c** Cross section of ureter resection specimen. **d** The metastasis to the ureter shows typical clear cell morphology

Surprisingly, the final histology showed a single metastasis of a clear cell renal carcinoma with a maximum diameter of 22 mm, in line with the initially diagnosed tumor of the left kidney (Fig. 4). No lymph node involvement was found.

The patient remained disease-free for 3 years but subsequently developed a secondary clear cell RCC within the inferior pole of the right kidney, with maximum diameter of 14 mm, which was successfully treated with a robotic-assisted partial nephrectomy. The follow-up with magnetic resonance imaging (MRI) at 6 months showed no tumor recurrence.

## Discussion

This case report presents a contralateral ureteral RCC metastasis in an otherwise healthy patient that was successful treated with partial ureterectomy and end-to-end anastomosis. Ureteral RCC metastasis is a very rare phenomenon, and to our knowledge, very few cases affecting the contralateral ureter are described in the literature [6]. Because of the rare and uncommon nature of the disease, the standard of treatment is not well established. Moreover, ureteral lesions carry the risk of renal failure in patients with, in most cases, (functionally) a single kidney and already reduced kidney function.

RCC metastasis can occur virtually anywhere, occasionally at very uncommon sites [4]. A standard approach for each possible metastatic site is not defined, and only low-evidence studies have been published. There are no randomized clinical trials evaluating the role of complete metastasectomy in metastatic RCC. A systematic review, summarizing the role of surgical metastasectomy and surgical outcomes for common sites of metastasis, found that a small subgroup of patient may benefit from aggressive surgical treatment. Better outcomes were associated with good performance status, long disease-free interval and limited burden of disease, ideally in the case of a single metastasis in a non-vital organ. However, the studies analyzed did not include ureteral metastasis [11].

The current recommendation of the European Association of Urology is to perform metastasectomy (with the exception of bone and brain metastases, for which radiotherapy can be offered as symptomatic treatment), especially in patients with favorable disease factors and where complete resection is achievable [1].

Different surgical techniques can be used to remove the ureteral metastasis. In our case, the mobility of the ureter was sufficient to permit free-tension anastomosis, and frozen section showed negative surgical margins. Therefore, we were able to perform a partial ureterectomy and end-to-end anastomosis with good functional outcomes. Other surgical techniques such as ileal ureteral substitution or Boary-plasty could be considered depending on the localization and size of the lesion and bladder capacity [5, 6, 8].

In this patient, the initial biopsies showed no malignancy, and subsequently, due to the size progression, both diagnostic and therapeutic surgical excision was performed. In 2018, the CARMENA trial [Clinical Trial to Assess the Importance of Nephrectomy] showed the non-inferiority of sunitinib alone compared to cytoreductive nephrectomy before adjuvant therapy with sunitinib in patients with metastatic RCC [3]. In cases of initially diagnosed metastatic RCC, other treatments such as systemic therapy with targeted agents could be discussed in an interdisciplinary tumor board, especially in patients with poor performance status. However, a subgroup of patients with low metastatic volume, especially those with good performance status and a single metastasis, could benefit from an aggressive surgical treatment as shown in this patient.

## Conclusion

The diagnosis of a single contralateral ureteral metastasis of RCC is very rare. The treatment should aim to preserve the single kidney function, as nephroureterectomy would consequently lead to lifelong dialysis. Thus, metastasectomy seems to be the therapy of choice in low-volume metastatic patients.

## Data Availability

All data are available within the manuscript.

## Abbreviations

RCC: Renal cell carcinoma
